# CleanSurvival: automated data preprocessing for time-to-event models using reinforcement learning

**DOI:** 10.1186/s12911-026-03786-6

**Published:** 2026-09-24

**Authors:** Yousef Koka, David Selby, Gerrit Großmann, Kathan Pandya, Sebastian Vollmer

**Affiliations:** 1 German University in Cairo, New Cairo City, Egypt; 2 Data Science and its Applications, German Research Center for Artificial Intelligence (DFKI), Trippstadter Str. 122, 67663 Kaiserslautern, Germany; 3 University of Kaiserslautern–Landau (RPTU), Gottlieb-Daimler-Str., 67663 Kaiserslautern, Germany; 4 Saarland University, Campus, 66123 Saarbrücken, Germany

**Keywords:** Survival analyis, AutoML, Q-learning, Data cleaning, Time-to-event

## Abstract

**Background:**

Data preprocessing is often paid little attention in machine learning, despite its potentially significant impact on model performance. While automated machine learning pipelines are starting to recognise and integrate data preprocessing into their solutions for classification and regression tasks, this integration is lacking for more specialised tasks like time-to-event models for censored data. As a result, survival analysis not only faces the general challenges of data preprocessing but also suffers from the lack of tailored, automated solutions in this area.

**Method:**

To address this gap, this paper presents CleanSurvival, a reinforcement-learning-based solution for optimizing preprocessing pipelines, extended specifically for survival analysis. The framework can handle continuous and categorical variables. It builds upon Learn2Clean’s *Q*-learning to select which combination of data imputation, outlier detection and feature extraction techniques achieves optimal performance for a Cox, random forest, neural network or user-supplied time-to-event model. The Python package is available on GitHub: https://github.com/datasciapps/CleanSurvival.

**Results:**

Experimental benchmarks on real-world datasets show that the *Q*-learning-based data preprocessing can improve predictive performance relative to simple baselines, while runtime behaviour is condition-dependent and most clearly interpretable in the best-covered benchmark cells. Furthermore, a simulation study demonstrates effectiveness across different types and levels of missingness and noise.

**Conclusion:**

With an increase in the use of machine learning, it becomes important to generalise AutoML pipelines to a variety of models now present, including survival analysis. Tools like CleanSurvival, which integrate preprocessing for survival analysis, can make survival studies faster and easier to perform, while also yielding more robust results.

**Supplementary information:**

The online version contains supplementary material available at https://doi.org/10.1186/s12911-026-03786-6.

## Introduction

In the era of big data and machine learning (ML), the ability to extract meaningful insights from complex datasets is paramount. A critical step in this process is *data preprocessing*, which involves cleaning, transforming, and preparing raw data to be suitable for analysis. The quality of data preprocessing may significantly impact the performance and reliability of ML models [1], even for robust learning methods [2]. This is particularly important in the field of survival analysis, where the goal is to predict the time until an event of interest occurs, such as patient death, equipment failure or customer churn.

Survival analysis poses unique challenges due to the presence of censored data, where the event of interest has not yet been observed. However, it is also overlooked in the context of automated machine learning (AutoML) pipelines, which aim to streamline the ML development process by automating tasks such as algorithm selection, hyperparameter tuning and model evaluation—and increasingly incorporate data preparation as part of their pipelines [3–5]. There exist several tools focusing on preprocessing for classification and regression models e.g. [6–9]. In this paper, we introduce CleanSurvival, an automated data preprocessing framework tailored specifically for survival analysis.

CleanSurvival leverages reinforcement learning (RL) techniques, specifically *Q*-learning, to optimise decisions such as imputation of missing values, detection and handling of outliers and feature extraction for survival models. RL is a powerful approach for automated data preprocessing because it dynamically optimises pipeline steps by learning to maximise a reward function tied directly to model performance, ensuring data cleaning decisions are guided by their impact on survival predictions. The framework is designed to handle continuous and categorical variables, and can be used with a variety of time-to-event models, from classical methods to modern deep learning frameworks.

The framework, available as an open-source Python package, is demonstrated on several common survival analysis datasets, highlighting the sensitivity of predictive performance to data preprocessing steps and boasting improved predictive performance compared to standard approaches.

The article is organised as follows. Section “Background” provides an overview of data preprocessing, survival analysis, AutoML tools and *Q*-learning. Section “Methodology” describes the architecture of CleanSurvival and its features. Section “Experiments” presents the results of experimental evaluation of the framework on real-world datasets and Section “Discussion” discusses CleanSurvival in comparison to other upcoming tools in this field while Section “Limitations” highlights some limitations. Finally, Section “Conclusion” discusses the results and outlines future directions for research.

## Background

Data preprocessing involves cleaning, transforming and organizing raw data into a suitable format for analysis, and is an important step in the ML pipeline. Sub-tasks of data preprocessing include imputation or removal of missing values, detection and handling of outliers, variable selection, feature extraction and data transformations. These steps can have a profound downstream impact on classification performance [10] and model explanations [11].

However, the selection of appropriate preprocessing methods often requires a combination of domain knowledge, visual inspection and manual experimentation; it is also often poorly documented, whether in academic papers or computational notebooks [12, 13]. Some authors have even attempted to quantify the effect of preprocessing steps on model predictions independently of the dataset [14].

Automated data preprocessing has emerged to address these challenges [4, 7, 9, 15]. This approach uses algorithms and heuristics to automate various data cleaning and transformation tasks, reducing the need for manual intervention or iteration and potentially improving the efficiency and effectiveness of the preprocessing stage, ideally by learning from past cleaning tasks [16]. However, the field is still in its infancy.

### Survival analysis

Survival analysis, also known as reliability analysis or duration modelling, is a statistical method for analysing time-to event data. It is widely used in various fields, including medicine, engineering and social sciences. In survival analysis, the primary goal is to model the time until an event of interest occurs, such as death, disease progression, machine failure or customer churn. The survival function,

*S(t) = P(T > t),*

denotes the probability that the time of event (death), a random variable *T*, occurred later than a time *t*. A unique characteristic of time-to-event problems is censoring, or data points that are only partially observed, such as patients who survived up until the last observation time, at which point they were lost to follow-up or the study period ended [17].

Naïvely, one can treat survival analysis as either a regression or classification problem, but both approaches lead to a significant information loss. In the former case, one treats the observed survival time as a continuous outcome, and censored observations are either discarded or imputed, resulting in substantial reduction in sample size or bias introduced by oversimplified assumptions about the censoring process. In the latter case, one models survival—or not—in a predefined time window, reducing the problem to binary classification and losing granular information about event times [18].

Survival analysis models, such as the Cox proportional hazards model, Kaplan–Meier estimator and accelerated failure time models, are widely used in practice [17, 19]. These models estimate the hazard function, survival function, or survival probabilities over time, providing valuable insights into the relationship between covariates and survival outcomes.

Concordance indices like the C-index are widely used in the evaluation of survival models due to their simplicity and ease of interpretation [20]. The C-index evaluates the model’s ability to correctly rank individuals based on their risk of experiencing the event. A higher C-index indicates better discriminatory power.

However, concordance indices have significant limitations, due to their poor calibration and failure to consider the distribution of survival times as well as their ranks [21]. The Brier score is another metric in survival analysis which calculates the accuracies of the predicted survival probabilities at a given time point [22]. Other calibration metrics like Houwelingen’s *α* or D-calibration can also be used to measure how well survival probabilities align with observed event times [23]. They could be compared against a baseline model such as the Kaplan–Meier estimator. Weighted integrated survival log loss or integrated Brier score (also known as the integrated Graf score) are recommended scoring rules [24].

To account for censoring, we apply inverse probability of censoring weighting (IPCW). Let *G(t)* be the Kaplan–Meier estimate of the probability of not being censored at *t*. Then, the integrated Brier score (IBS) is defined

$$\mathrm{IBS} = \frac{1}{n} \sum_{i=1}^n \sum_{j=1}^k \Delta t_j \, \frac{\left( S(t_j | x_i) - \mathrm{Observed}(t_j, i) \right)^2}{G(t_j) }, $$

for a set of time points $\{t_1, t_2, \ldots, t_k\}$, where $\Delta t_j$ represents the difference between time points, *n* is the number of individuals and $\mathrm{Observed}(t, i)$ is an indicator function, equal to *1* if the individual is known to have survived beyond *t* and *0* otherwise.

### Automated machine learning

Automated machine learning (AutoML) aims to streamline the process of ML development by automating steps such as algorithm selection, hyperparameter tuning and model evaluation, reducing the amount of time and expertise required by practitioners to train, deploy and fine-tune models [25, 26]. AutoML frameworks have seen success in various applications by employing search strategies such as meta-learning, Bayesian optimisation and ensemble learning to achieve competitive performance.

However, data preprocessing remains an important analysis step that typically falls outside the AutoML pipeline [27]. Data preparation steps including cleaning, normalisation and feature engineering are critical for the success of ML models but can be highly problem-specific [28]. Automating these tasks while maintaining flexibility for diverse datasets remains a significant hurdle [4, 15]. Mahdavi et al. [16] highlighted the potential of AI to solve data quality problems through data profiling and learning from past cleaning attempts [29]. A holistic approach integrates the cleaning process with downstream tasks so that the cleaning is optimised for predictive performance [30]; indeed when integrated into an AutoML framework, some cleaning steps may be more important than others [31].

Survival analysis in particular faces particular challenges, partly due to the relative lack of support for such models in the first place (versus classification or regression), as well as unique difficulties of handling censored time-to-event data [17], which are typically not addressed in conventional AutoML frameworks and do not feature in AutoML surveys [25]. Seamlessly integrating these domain-specific preprocessing steps with the downstream tasks of model optimisation and evaluation is a complex, underexplored area.

Prominent AutoML pipelines include Auto-WEKA, an early AutoML system that uses Bayesian optimisation to search for the best combination of preprocessing steps and machine learning algorithms [32]. It covers a range of models but does not cover crucial survival models like Cox or survival metrics [33]. TPOT is a tree-based pipeline optimisation tool that uses genetic programming to evolve pipelines of data cleaning and machine learning operations, however it performs simple imputation and does not perform censoring natively, thus cannot work on survival analysis [34]. Similarly, auto-sklearn (an extension of Auto-WEKA that incorporates more recent advancements in machine learning and hyperparameter optimisation, while offering a familiar interface based on the Python package scikit-learn) cannot perform preprocessing for survival models and metrics [26].

Salhi et al. [15] presented a recent survey of data preprocessing using AutoML (though survival analysis is not mentioned). In their review, they highlight the relative capabilities of AutoML platforms: in many cases the data processing support is relatively basic. All 11 tools reviewed support missing value imputation, though for specific frameworks like auto-sklearn, certain types of missing values must still be handled manually by the user before the automated pipeline can proceed. In such cases, claims of support for automated data processing may reflect the capabilities of the underlying ML framework (i.e. scikit-learn) rather than fully autonomous handling. Mumuni and Mumuni [4] also surveyed automated data processing for deep learning applications, highlighting in more detail the extent to which data processing steps are integral components of the automated pipeline. They similarly note the lack of early support from auto-sklearn.

Table 1 compares the features of various AutoML solutions. Of frameworks offering automated data cleaning, DataRobot is a proprietary, commercial platform that models time-to-event data by converting the task into a classification problem via time discretisation also called ‘survival stacking’ [35]. Google AutoML Tables, Amazon SageMaker Autopilot and Azure ML can perform different preprocessing steps, but they are not always tailored to survival analysis and these are all proprietary tools [36]. Big ML, which performs decision tree-based optimisation, enables preprocessing but not survival analysis [37]. MLJAR and AutoGluon both use stack ensembling, and perform most of the preprocessing steps needed but do not support survival analysis [38, 39]. PyCaret can perform preprocessing for regression or classification, but cannot handle censored time-to-event data without external tools [40]. H2O.ai can run Cox proportional hazards models [41] as a fixed model, but not via its AutoML interface as it requires advanced platforms for the same.

**Table 1:** Comparison of AutoML frameworks with respect to data preprocessing and survival analysis capabilities

Framework	License	Missing values	Categorical	Imbalance	Survival
auto-sklearn	Open-source	Requires NaN removal	Yes	Yes	Externallibraries
TPOT	Open-source	Yes	No	No	No
Google AutoML Tables	Proprietary	Yes	Yes	Yes	No
H2O AutoML	Open-source	Yes	Yes	Yes	Limited
PyCaret	Open-source	Yes	Yes	Yes	Limited
AutoKeras	Open-source	Yes	Yes	Limited	Custom models
Azure AutoML	Proprietary	Yes	Yes	Yes	External tools
BigML	Commercial	Yes	Yes	Limited	No
MLflow	Open-source	No	No	No	No
FLAML	Open-source	No	No	Yes	No
MLJAR	Open-source	Yes	Yes	Limited	No
DataRobot	Proprietary	Yes	Yes	Yes	Limited
Amazon SageMaker Autopilot	Proprietary	Yes	Yes	Yes	No
AutoGluon	Open-source	Yes	Yes	Limited	External libraries

However, some dedicated data cleaning solutions have been proposed. Bilal et al. [7] proposed Auto-Prep, an interactive Python-based tool that recommends data cleaning methods to the user based on application of candidate techniques and subsequent evaluation using simple classifiers or regression models. In their accompanying review of data preprocessing in AutoML, Bilal et al. [7] highlight the widespread lack of capabilities among existing tools to perform data preprocessing and feature engineering without manual human intervention. Another Python package, Atlantic [9], automates preprocessing steps including feature engineering and missing value imputation for supervised learning tasks. The framework identifies the best combination of steps based on evaluation using tree-based model ensembles.

MLsurvival [42] described an automated tool for cancer survival prediction that removes or imputes missing values, selects and standardises features, trains survival models and then makes predictions. Unfortunately, neither a full text article nor open source implementation of the method were published. More recently, Pomsuwan and Freitas [43] proposed an AutoML system for survival analysis based on genetic algorithms and a combination of elastic-net Cox models, random survival forests and survival trees, optimizing for C-index. However, the tool does not incorporate data preprocessing. Other frameworks like AutoPrognosis [44] have developed automated tools using Bayesian optimisation that include survival analysis, though with less scope for customisation of preprocessing pipelines. The widely used library scikit-survival integrates scikit-learn preprocessing with survival models, but it requires manual pipeline building [45]. The R package mlr3proba provides a machine learning interface for survival analysis and preprocessing, however missingness is dealt with externally [46].

### *Q*-learning

Reinforcement learning is well-suited to the task of automating constrained ML pipelines, as it optimises sequential decision-making processes, balancing exploration and exploitation, while being less computationally intensive than other methods, such as unconstrained evolutionary algorithms [47].

*Q*-learning [48] is a model-free off-policy reinforcement learning algorithm that seeks to find the optimal action-selection policy for an agent interacting with an environment. The algorithm is based on estimating the value *Q=Q(s, a)* of taking an action *a* in a given state *s*. The agent iteratively updates these *Q* values based on its experiences, enabling it to learn an optimal policy even in environments with stochastic rewards and transitions.

The goal of *Q*-learning is to maximise the cumulative reward over time by updating *Q* according to the Bellman equation. Given a current state *s*, action *a*, reward *r* and next state $s^\prime$, the update rule is:

1 $$Q(s, a) \leftarrow Q(s, a) + \alpha \bigl( r + \gamma \max_{a^\prime} Q(s^\prime, a^\prime) - Q(s, a) \bigr),$$

where *α* is the learning rate and *γ* is the discount factor, controlling the importance of future rewards. The update Equation Eq. 1 allows the *Q*-learning agent to converge to an optimal policy $\pi^*$, defined

2 $$\pi^*(s) = \arg\max_a Q(s, a).$$

without requiring a model of the environment’s dynamics. By exploring various state–action pairs and refining *Q*-values, *Q*-learning is able to asymptotically approach optimal behaviour, provided that the agent balances exploration and exploitation effectively.

Alternatives to *Q*-learning, such as Bayesian optimisation, can offer improved sample efficiency in some cases but often struggle to scale in high-dimensional or discrete search spaces typical of complex AutoML pipelines [6, 49]. More specialised methods, such as neural architecture search, are not easily extensible to data preprocessing tasks. Other reinforcement learning approaches, including deep reinforcement learning [47] and Monte Carlo tree search [50], provide flexibility but introduce additional computational overhead and may require constraints or tailored mechanisms to ensure valid ML pipelines.

Berti-Equille [6] developed Learn2Clean, a tool offering an innovative approach to data preprocessing. It leverages *Q*-Learning to dynamically select the optimal sequence of preprocessing tasks for a given dataset and ML model. This optimisation aims to maximise the quality of the ML model’s results. Learn2Clean implements automated preprocessing for regression, classification and clustering tasks, using RL to optimise respective evaluation criteria: mean squared error, accuracy and silhouette index. However, the framework lacks built-in support for time-to-event data, categorical data types, flexible hyperparameter tuning and custom reward functions. It also has a complex dependency structure, which can make initial setup challenging for end-users. We build upon Learn2Clean, by adapting the reinforcement learning framework to survival analysis.

## Methodology

**Fig. 1:**
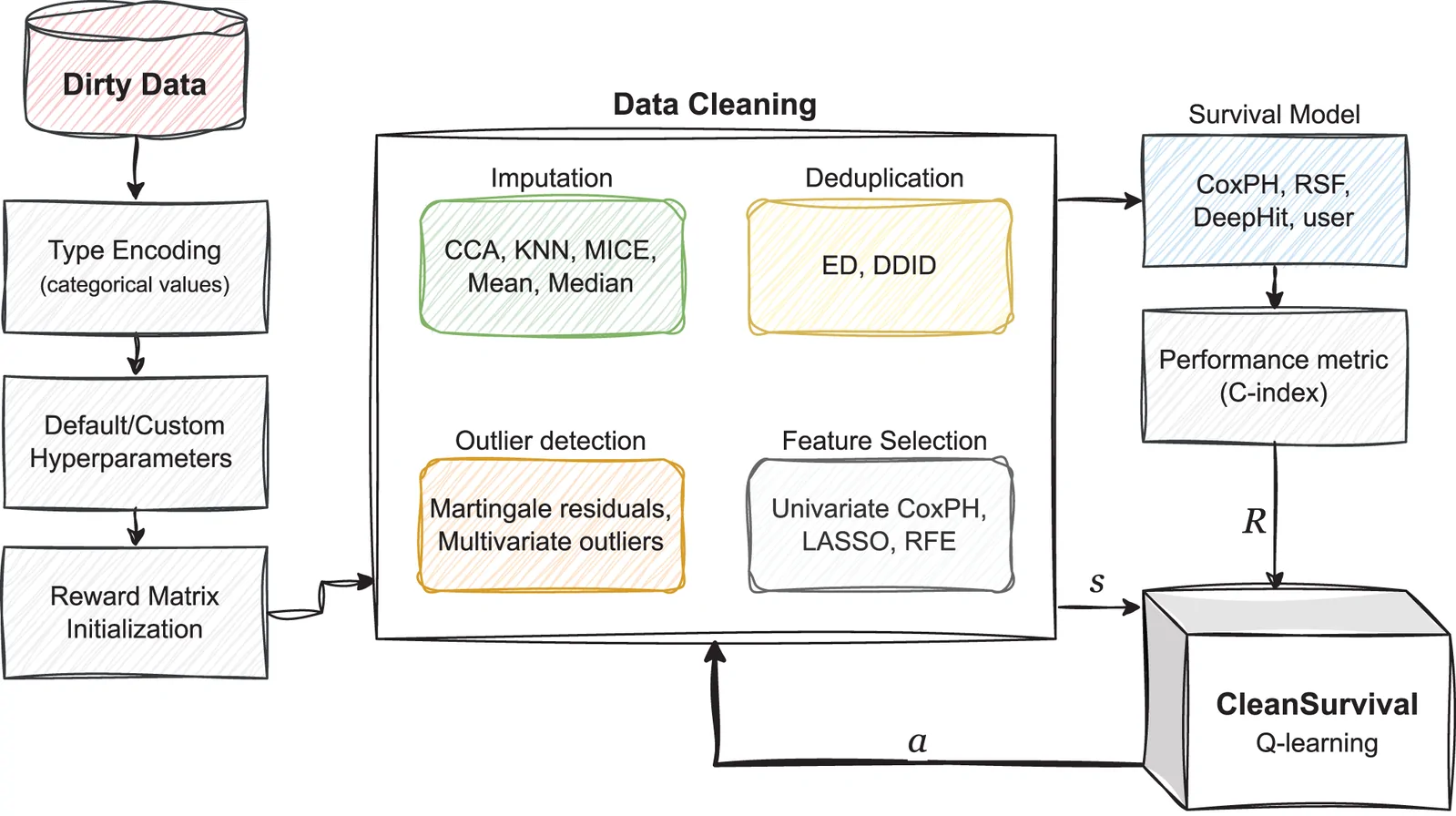
Architecture of the cleansurvival automated data cleaning framework

In this section we describe CleanSurvival, our proposed AutoML data preprocessing tool for survival analysis, illustrated in Fig. 1.

### Data preprocessing methods

#### Missing values

CleanSurvival offers a variety of methods for handling missing values, addressing different data characteristics and analytical goals.

- For straightforward scenarios with minimal missingness, the complete case analysis (CCA) simply removes rows containing missing values.
- Simple mean/median imputation replaces missing values with the mean or median of the observed values for a given variable.
- Multiple imputation using chained equations [51] provides a more robust approach by generating multiple suitable replacements for each missing value, creating several complete datasets for analysis. This method utilises an iterative imputer, which starts with an initial guess and refines the estimates until convergence.
- Finally (and most computationally intensive): *k*-nearest neighbors (KNN) imputation identifies the *k* most similar observations to the one with missing values, based on other, non-missing features, and uses their values to impute the missing data. Mean or median imputation is a limit case where *k→ n*.

#### Outliers

To ensure the reliability and validity of survival analysis, robust outlier detection methods are essential [52].

- For multivariate datasets, CleanSurvival employs the Elliptic Envelope algorithm [53] to identify and remove outliers based on their Mahalanobis distances from the data centre. This method is particularly useful for detecting outliers that deviate from the overall correlation structure of the data.
- The Martingale residuals method [54] calculates the difference between the observed and expected number of events for each individual (based on a simple Kaplan–Meier estimator), providing a measure of how unusual their survival time is compared to the expected survival time.

#### Variable selection and feature extraction

To identify the most salient variables for survival analysis, a variety of feature selection methods are available, enhancing both the model performance and interpretability.

- The Univariate Cox Proportional Hazards Selection (UC) method assesses the individual effect of each feature on survival using the Cox Proportional Hazards model. It selects features based on the significance of their coefficients, highlighting the variables strongly associated with survival outcomes.
- The LASSO (Least Absolute Shrinkage and Selection Operator) regression technique shrinks the coefficients of less important features to zero, effectively performing feature selection.
- Recursive Feature Elimination (RFE) recursively removes the least important features based on their contribution to a model’s performance, using cross-validation to evaluate the model’s performance at each step.
- The Information Gain Selection (IG) method measures the amount of information gained about the target variable (survival outcome) by knowing the value of a feature. This helps identify the most relevant variables by selecting features that provide the most information about the survival outcome.

### Survival analysis

Three survival analysis models were carefully selected to integrate into CleanSurvival, each chosen for its unique strengths and applicability to a variety of survival analysis scenarios.

*Cox proportional hazards* This widely-used model [55] is valued for its interpretability, allowing researchers to quantify the impact of different factors on the hazard rate. The core assumption of this model is that the hazard ratio between any two individuals is constant over time (the proportional hazards assumption). While violations of this assumption can lead to biased or uninterpretable estimates in standard analysis, our pipeline search explicitly optimises for the downstream C-index. Consequently, the chosen preprocessing transformations (such as feature selection or imputation) may indirectly help mitigate severe violations.

*Random survival forest* The RSF model [45] is an ensemble method based on decision trees, offering robustness to nonlinearities and interactions in the data. Its non-parametric nature makes it a flexible choice when the underlying relationships in the data are not well understood.

*DeepHit neural network* This deep learning model [56] leverages the power of neural networks to capture complex patterns and interactions in survival data. It is a specialised form of deep neural network, which, instead of a single risk score, estimates a discrete probability mass function over fixed time intervals. Its ability to model multiple competing risks makes it particularly well-suited for scenarios where individuals may experience different types of events.

#### Reward structure

To guide the *Q*-learner effectively for survival analysis problems, the reward structure uses the concordance index (C-index) on a hold-out set [20]. Because this reward is evaluated directly on the downstream survival model’s predictions, the *Q*-learning agent identifies preprocessing pipelines that are optimised for the chosen model class. This means that a preprocessing pipeline that is optimal for a Cox proportional hazards model may differ from that optimised for a random survival forest.

### Working modes

To provide users with a range of options for data preprocessing and analysis, four distinct working modes were implemented in CleanSurvival:

*Main algorithm* This mode uses the core *Q*-learning algorithm to identify the optimal sequence of preprocessing steps that maximises the performance of the chosen survival analysis model. This is the primary mode for automated pipeline optimisation.

*Random cleaning* In this mode, users can specify the desired number of random experiments. The tool generates random preprocessing pipelines and evaluates their performance, providing insights into the impact of different preprocessing choices. This mode can serve as a baseline for comparison with the optimised pipeline.

*Custom pipeline* This mode allows users to define their own fixed preprocessing pipelines using a simple text configuration file. Each line in the file specifies a sequence of preprocessing methods, providing flexibility for testing specific hypotheses or domain knowledge.

*No preparation* This mode bypasses all preprocessing steps, directly passing the raw dataset to the chosen survival analysis model. This can be useful for establishing a baseline for performance without any preprocessing.

The inclusion of these working modes significantly enhances the utility of the framework by offering baselines for evaluating the effectiveness of the optimised pipelines generated by the *Q*-learning algorithm.

## Experiments

### Experimental setup

All methods were implemented in Python. Source code and documentation are available at https://github.com/datasciapps/CleanSurvival.

Details of the datasets used are provided in Table 2. We demonstrate the approach using the rotterdam dataset, derived from the Rotterdam Study [57], a large prospective cohort study in the Netherlands, flchain [58], a study of the relationship between serum free light chain (a type of blood measurement) and mortality, and gbsg [59], containing data from the German Breast Cancer Study Group. These datasets are distributed as part of the R package survival [60, 61]. The flchain dataset contains naturally occurring missing values, for the ‘creatinine’ and ‘chapter’ variables of 17.15% and 72.45% respectively, while the rotterdam and gbsg datasets do not contain any missing values, allowing us to simulate different types and levels of missingness for benchmarking purposes.

**Table 2:** Summary of datasets used in the experiments

Dataset	Samples	Features	Source
Rotterdam	2982	14	Royston and Altman [57]
Flchain	7874	11	Dispenzieri et al. [58]
GBSG (German Breast Cancer Study Group)	686	8	Schumacher et al. [59]

The results of data preprocessing strategies suggested by CleanSurvival are compared against the following methods:

1. Complete case analysis (CCA): effectively ignoring the problem of missingness and outliers
2. Random selection of imputation methods: simulating a grid search over possible analysis choices
3. Mean imputation, as an example of a reasonable baseline used by an analyst not exploring the sensitivity of the model to different imputation strategies

We evaluate each method based on predictive performance metrics (C-index, IBS) as well as the computational time taken to retrieve a performant pipeline (defined as reaching within 5% of the final best-observed C-index for that dataset).

For model evaluation, the current Cox and random survival forest wrappers use an 80:20 hold-out split (test_size = 0.2).

Hyperparameters that are not part of the CleanSurvival search space were left at their default values for the scikit-survival models: the Cox model was fit with default penalisation and the RSF was trained with 100 estimators and a minimum of 3 samples per leaf. The architectural choices for the DeepHit deep learning framework were as follows: the architecture consists of a shared sub-network with two hidden layers, each containing 100 neurons, using ReLU as the activation function. This is followed by cause-specific sub-networks, each containing two hidden layers with 50 neurons per layer. Dropout regularisation was applied to all layers with a keep probability of 0.8 to mitigate overfitting. The model was trained using the Adam optimiser with a learning rate of 0.001 for 10,000 iterations. In practice, these options are customizable by the user or could be incorporated into the reinforcement learning action space as additional modules.

The Rotterdam dataset does not contain missing values, so synthetic missingness was introduced using the Jenga framework [62]. Three missingness mechanisms were simulated across multiple variables: missing completely at random (MCAR, where missingness is independent of any variable), missing at random (MAR, where missingness depends only on fully observed variables like age or treatment group), and missing not at random (MNAR, where missingness depends on the unobserved value itself, simulating the unrecorded worst-case outcomes). We evaluated these mechanisms at missingness levels ranging from 10% to 50%. After generating pipelines using CleanSurvival and random search, we computed the integrated Brier scores (IBS) across dataset variations.

### Results

To evaluate optimisation dynamics and calibration clearly, we focus on pipelines optimised for the Cox proportional hazards model. This keeps convergence and proportional-hazards diagnostics interpretable within a single model family. DeepHit results are not shown because intensive neural architecture search/training inside the reinforcement-learning loop is beyond the scope of this paper.

Table 3 compares survival models fitted to GBSG, Flchain and the three 50% Rotterdam missingness settings. In these summaries, CleanSurvival remains stronger than the simple analyst baselines (CCA and mean imputation) in the main Rotterdam 50% settings and on Flchain.

**Table 3:** Predictive performance of survival models, measured by C-index, for 50% missingness benchmark configurations of Rotterdam/GBSG and natural missingness for Flchain. Mechanism indicates the type of synthetic missingness missing at random, missing completely at random or missing not at random. Entries report mean C-index *±* standard error over 50+ runs

Dataset	Mechanism	CCA	CleanSurvival	Mean	Optuna	Random
Flchain	None	*0.395 ± 0.011*	*0.660 ± 0.001*	*0.617 ± 0.002*	*0.656 ± 0.000*	*0.737 ± 0.020*
Rotterdam	MAR	*0.772 ± 0.005*	*0.811 ± 0.002*	*0.787 ± 0.004*	*0.565 ± 0.051*	*0.828 ± 0.002*
Rotterdam	MCAR	*0.789 ± 0.004*	*0.824 ± 0.003*	*0.793 ± 0.004*	*0.614 ± 0.041*	*0.824 ± 0.001*
Rotterdam	MNAR	*0.789 ± 0.004*	*0.810 ± 0.002*	*0.783 ± 0.004*	*0.625 ± 0.046*	*0.858 ± 0.007*
GBSG	MNAR	–	*0.632 ± 0.004*	–	*0.650 ± 0.000*	*0.684 ± 0.008*

To verify that this pattern is not specific to Cox proportional hazards models, results for RSF models in similar scenarios are provided separately in the supplementary material.

Feature-selection operators were frequently selected by the Q-learning policy: LASSO appeared in 74/109 (67.9%) CleanSurvival pipelines and RFE appeared in 46/109 (42.2%). This suggests that CleanSurvival achieves performance gains through a policy of dynamically filtering out noisy features to stabilise the downstream survival models.

**Fig. 2:**
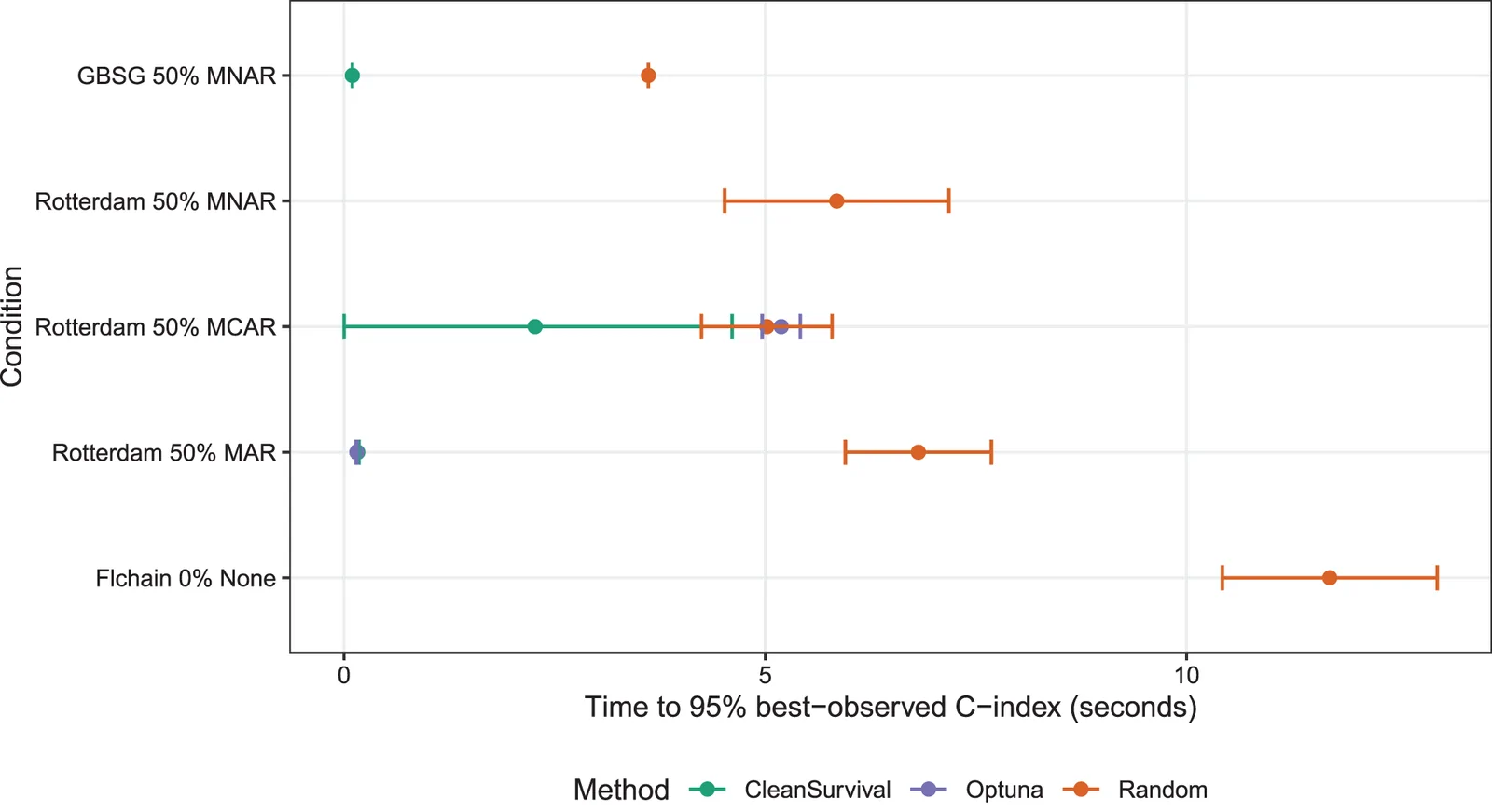
Runtime to target (time to reach 95% of best-observed C-index) across selected benchmark settings. Points show medians, horizontal bars show IQR

In Flchain, random search occasionally found a better edge case, as it is a high-variance unconstrained exploration, compared to the more narrowly controlled search of CleanSurvival, which has significantly reduced runtime as shown in Fig. 2.

In Table 4, we report Wilcoxon signed-rank contrasts for paired experimental runs.

**Table 4:** Wilcoxon summary (CleanSurvival vs Optuna/Random) for the selected C-index benchmark. Rotterdam and GBSG rows correspond to 50% missingness settings (by synthetic missingness mechanism: missing at random, missing completely at random or missing not at random), while Flchain uses its natural missingness. *p*-values are Holm-adjusted across all evaluated conditions

Dataset	Mechanism	Comparator	*Δ*	Win rate	*p*
Flchain	None	Optuna	−0.00	0.48	1.49e-02
Flchain	None	Random Search	−0.01	0.39	4.04e-02
Rotterdam	MAR	Optuna	0.80	0.68	3.54e-05
Rotterdam	MAR	Random Search	−0.02	0.29	3.42e-03
Rotterdam	MCAR	Optuna	0.80	1.00	2.18e-08
Rotterdam	MCAR	Random Search	−0.00	0.50	2.29e-01
Rotterdam	MNAR	Optuna	0.80	0.66	7.30e-05
Rotterdam	MNAR	Random Search	−0.03	0.22	1.20e-05
GBSG	MNAR	Optuna	−0.02	0.02	6.14e-09
GBSG	MNAR	Random Search	−0.04	0.03	3.94e-04

Figure 2 summarises runtime behaviour. The complete numeric runtime table summarizing the distributions across conditions is provided in the supplementary material.

**Fig. 3:**
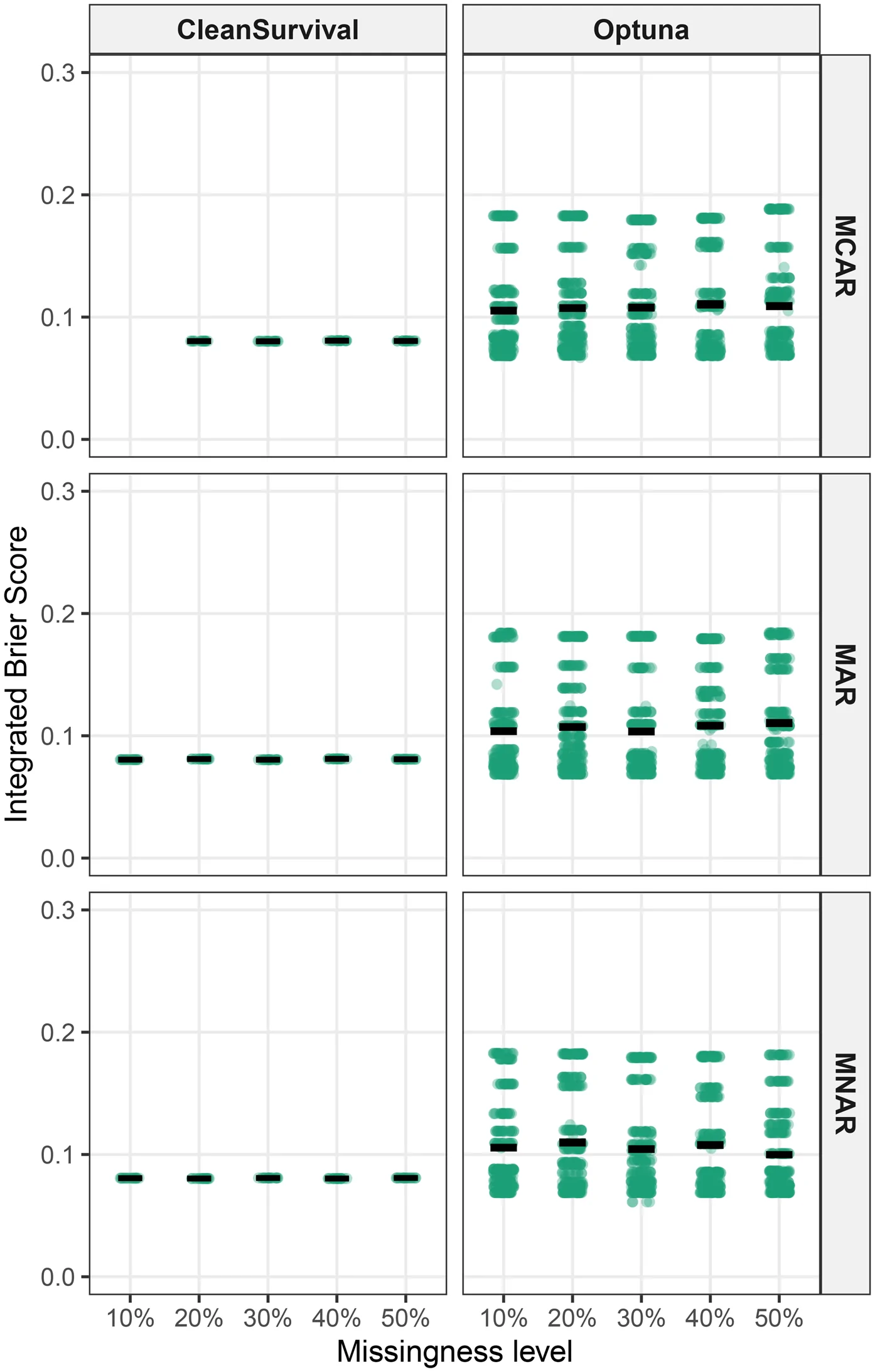
Integrated Brier Score (IBS) for Q-learning and Bayesian optimisation on Cox model pipelines with the Rotterdam dataset across different missingness structures. Each point represents a single evaluated pipeline. Crossbars show mean *±* SE. Lower is better

Figure 3 demonstrates that CleanSurvival yields highly stable, low-variance performance in integrated Brier Score across all missingness structures. In contrast, Optuna exhibits significant variance apparently due to unstable calibration in unconstrained pipeline optimisation.

**Fig. 4:**
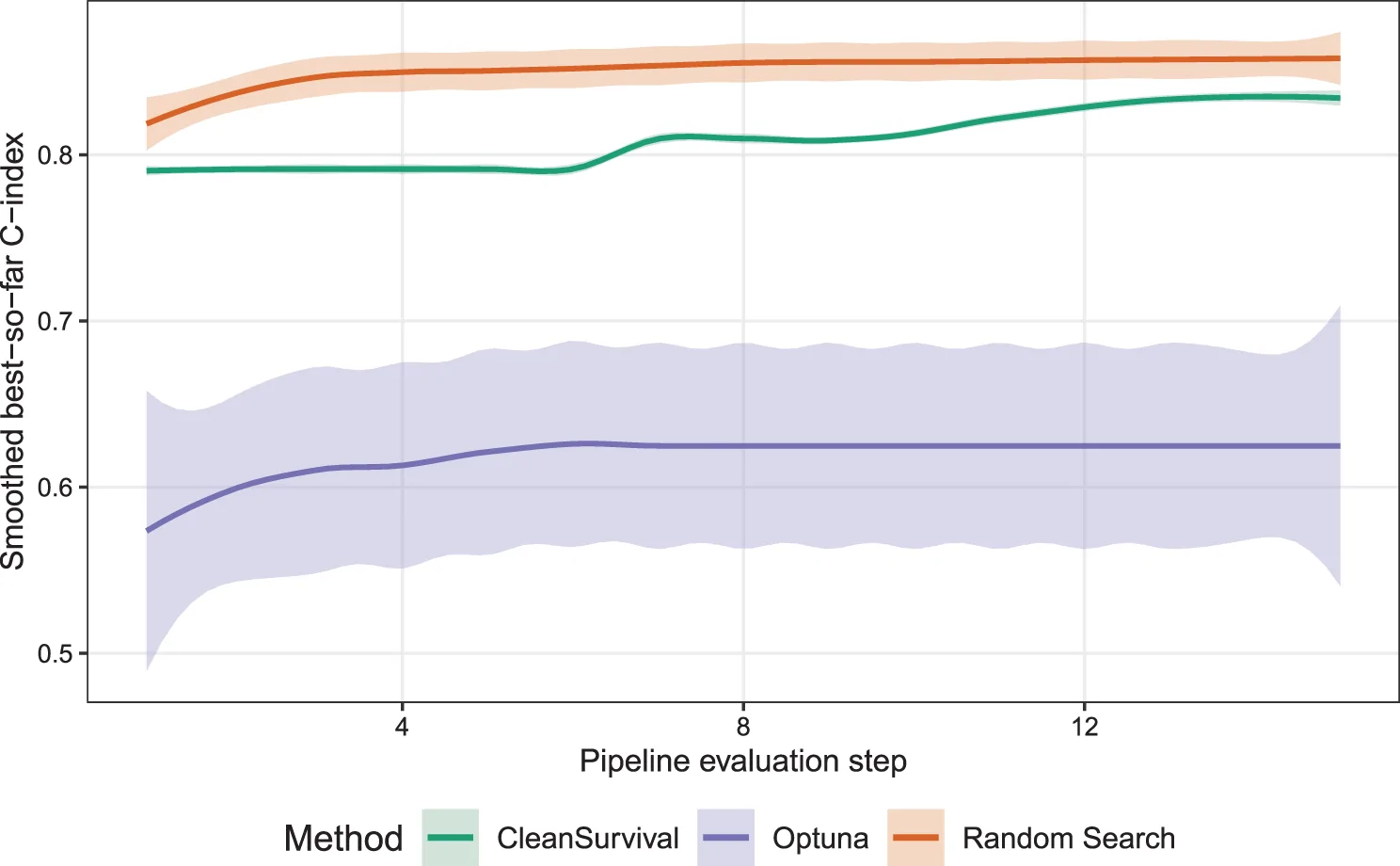
Aggregate convergence (step) for Rotterdam at 50% MNAR. Curves show LOESS-smoothed best-so-far C-index trajectories for CleanSurvival, Optuna, and Random Search

Figure 4 shows the step-based aggregate convergence (the average best-historical C-index at each sequential RL iteration). Traces are smoothed using locally estimated scatterplot smoothing (LOESS) to illustrate the learning trajectory.

**Fig. 5:**
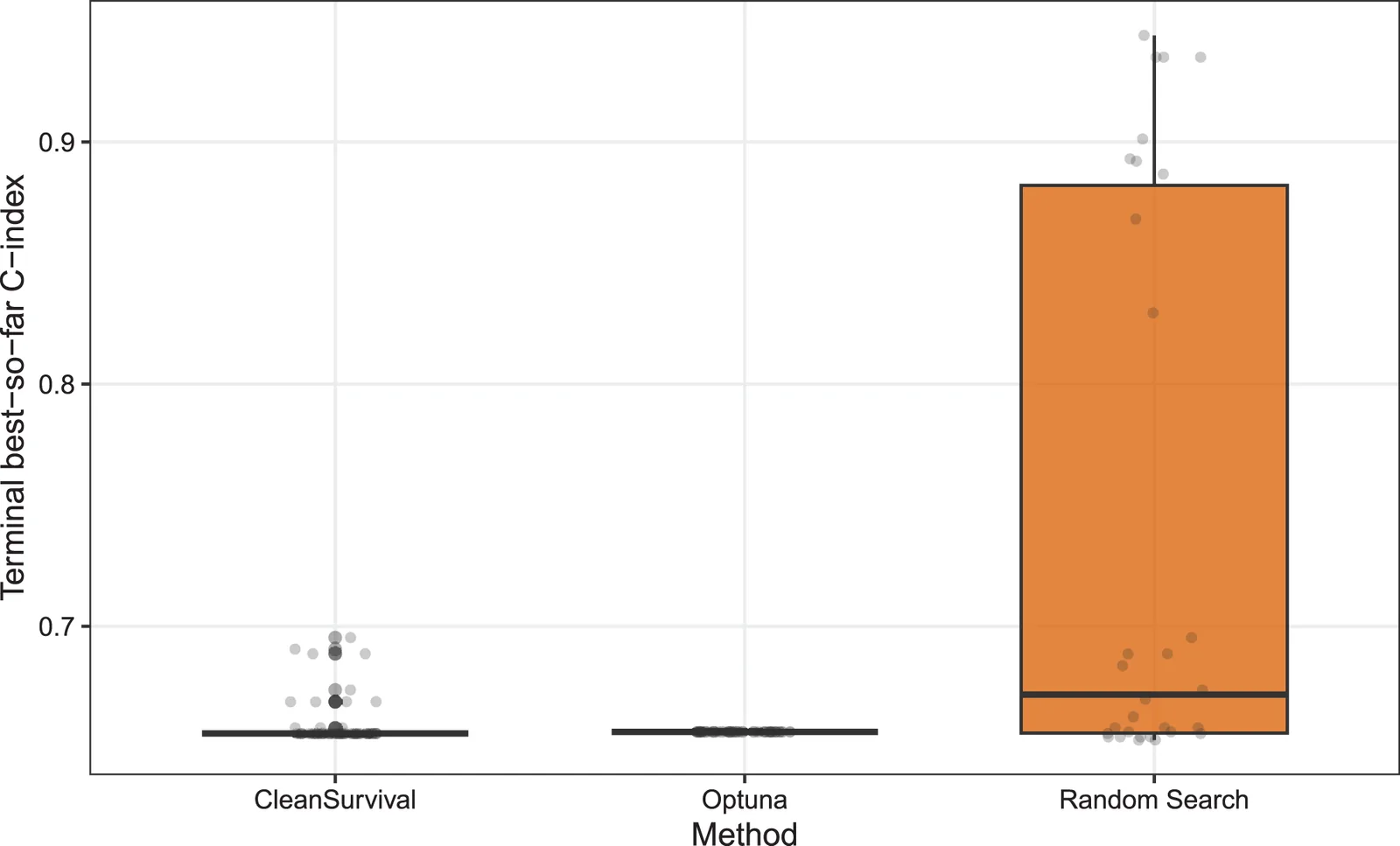
Algorithm terminal performance on Flchain (C-index). Each box summarises the per-run terminal best-so-far C-index; unknown method labels are excluded

Figure 5 summarises the terminal best-so-far C-index per run, excluding a marginal fraction of runs compromised by telemetry logging errors.

**Fig. 6:**
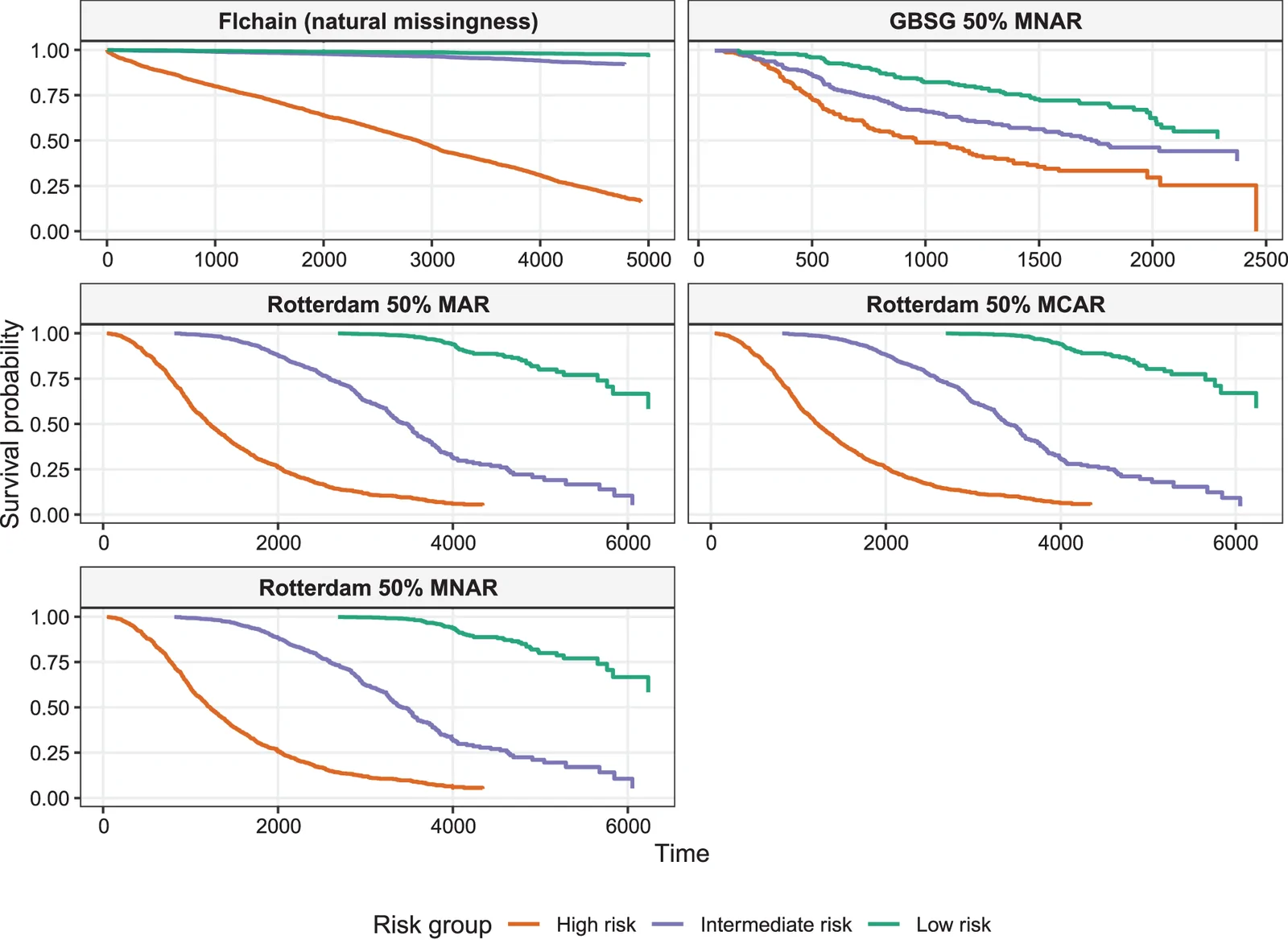
Kaplan–Meier survival curves by model-derived risk stratum across key benchmark conditions. For each condition, a baseline Cox model is fit using simple median imputation applied within that specific missingness scenario, subjects are divided into low/intermediate/high risk tertiles by linear predictor, and observed Kaplan–Meier curves are plotted per stratum

Figure 6 demonstrates CleanSurvival’s clinical utility. By successfully stratifying patients into distinct low, intermediate, and high-risk groups, it shows that the automated preprocessing preserves underlying survival signals. Diagnostic plots of Schoenfeld residuals are provided in the supplementary material.

## Discussion

With most of the AutoML preprocessing tools focusing on classification and regression models, we present CleanSurvival as a tool which builds upon Learn2Clean to tailor preprocessing for survival analysis using a *Q*-learning-based framework and reward structures. Survival analysis is important in fields like cancer research, and with the abundance of machine learning algorithms, it becomes crucial to develop automated approaches that can handle the unique challenges of censored time-to-event data [63]. Our results demonstrate that integrating data cleaning directly into the optimisation loop—guided by survival-specific predictive performance metrics—yields pipelines that are adapted to the downstream model.

The Integrated Brier Score (IBS) evidence indicates that calibration is broadly stable across most missingness settings for the Cox model, with the exception of the 40% MCAR condition, which exhibited higher variance.

This makes CleanSurvival a valuable flexible tool in the emerging field of automation for machine learning and preprocessing pipelines, capable of supporting various survival models, including neural networks, while permitting user customisation.

In a realistic, practical scenario—such as when analysing high-dimensional electronic health records—practitioners often struggle to choose the best sequence of imputation, transformation, and feature selection strategies [64]. Rather than manually testing various missingness imputation rules or arbitrary variable selections, a clinical data scientist can configure CleanSurvival with their chosen downstream analysis method alongside the provided dataset. The framework automates the hyperparameter combinations and step-order decisions. The optimised outputs are fully compatible with external libraries; the extracted data transformations from the final best pipeline can be seamlessly serialised and utilised within scikit-survival’s scikit-learn compatible wrapper or passed on to subsequent downstream architectures like those found in AutoPrognosis.

Furthermore, integrating diagnostic tools early in the automated process is necessary for clinical reporting. It allows quicker analysis of data and can allow better translation of results into clinical practice [65]. As outlined in the methods and empirical derivations in the Appendix, the optimisation process itself tends to select pipelines which naturally stabilise model assumptions on average, such as keeping proportional hazards relatively constant for the Cox model. By filtering noisy features out via feature reduction, CleanSurvival inherently guides the final model towards stability in complex, high-missingness regimes.

## Limitations

Several limitations must be acknowledged. First, although the analysis includes three datasets, the generalisation of these performance gains to higher-dimensional or multi-modal clinical datasets remains unverified. Second, the current implementation has computational overhead, depending on the configurations selected, especially when utilizing *k*-nearest neighbors imputation and complex deep learning survival models repeatedly alongside the *Q*-learning iterations. Isolating the marginal benefits of specific search-space subsets via ablation studies is left for future work.

## Conclusion

In this paper, we have introduced CleanSurvival, a *Q*-learning-based framework that automates data preprocessing for survival analysis, addressing the often underserved challenge of automation in the presence of censored time-to-event data. We demonstrated its ability to learn performant preprocessing pipelines across different settings, compared to complete case analysis, mean imputation, and undirected random search.

The framework’s adaptability to various survival analysis models and its support for diverse preprocessing techniques make it a valuable tool for researchers and practitioners. Experimental results confirm that CleanSurvival can facilitate the discovery of optimal pipelines and maintain robust performance under different missingness patterns, though comparative runtimes remain heavily condition-dependent. These findings underscore the critical role of automated data preprocessing in enhancing the reliability of survival models and the feasibility of integrating reinforcement learning techniques into AutoML workflows.

Future work will focus on extending the framework to handle additional preprocessing tasks, incorporating advanced reinforcement learning strategies and improving scalability for large datasets and complex pipelines. Additionally, whether ensembling over differently cleaned datasets, together with raw data, can increase predictive performance or instead increase data-processing bias, is an open question for future research.

## Electronic supplementary material

Below is the link to the electronic supplementary material.

Supplementary Material 1

## Data Availability

All datasets and code used in this study are publicly available. The analysis code and processed data required to reproduce the results and figures are hosted at https://github.com/datasciapps/CleanSurvival. The primary datasets analysed are publicly available from the R survival package (CRAN) and the original studies as listed below: Rotterdam (Rotterdam Study): distributed as part of the R survival package (CRAN) --- https://cran.r-project.org/web/packages/survival/survival.pdf. Original source: Royston_Rotterdam_2013. Flchain (free light chain study): dataset flchain in the R survival package (CRAN) --- https://cran.r-project.org/web/packages/survival/survival.pdf. Original source: Dispenzieri_FLchain_2012. GBSG (German Breast Cancer Study Group): dataset gbsg as distributed via the R survival package (CRAN) --- https://cran.r-project.org/web/packages/survival/survival.pdf#page=59%26zoom=100,133,776. Original source: Schumacher1994. These datasets do not have individual accession numbers; their stable location is the CRAN survival package, and the original cohort descriptions are provided in the cited references.
