# Supplementary material for: CleanSurvival: automated data preprocessing for time-to-event models using reinforcement learning
Source: BMC Med Inform Decis Mak. 2026 Sep 24;26:367. doi: 10.1186/s12911-026-03786-6 (PMC13613583; doi:10.1186/s12911-026-03786-6)
Supplement: Supplementary file 1 [file 12911_2026_3786_MOESM1_ESM.pdf]

Appendix: CleanSurvival

1 Runtime Summary Table

Table 1: Observed run counts (n) for comparison cells in the main paper.

Dataset	Mechanism	Method	n_runs
Flchain	None	CCA	431
Flchain	None	CleanSurvival	16
Flchain	None	Mean	408
Flchain	None	Optuna	50
Flchain	None	Random	31
Rotterdam	MAR	CCA	360
Rotterdam	MAR	CleanSurvival	20
Rotterdam	MAR	Mean	360
Rotterdam	MAR	Optuna	59
Rotterdam	MAR	Random	20
Rotterdam	MCAR	CCA	360
Rotterdam	MCAR	CleanSurvival	44
Rotterdam	MCAR	Mean	360
Rotterdam	MCAR	Optuna	81
Rotterdam	MCAR	Random	20

Table 1: Observed run counts (n) for comparison cells in the main paper.

Dataset	Mechanism	Method	n_runs
Rotterdam	MNAR	CCA	360
Rotterdam	MNAR	CleanSurvival	28
Rotterdam	MNAR	Mean	360
Rotterdam	MNAR	Optuna	68
Rotterdam	MNAR	Random	30
GBSG	MNAR	CleanSurvival	2
GBSG	MNAR	Optuna	50
GBSG	MNAR	Random	30

Table 2: Comparisons of random survival forests for selected scenarios (Flchain natural missingness, Rotterdam 50% MNAR, GBSG 50% MNAR). Values are mean ± standard error of best-observed C-index per rerun; each cell has 10 reruns.

Dataset	Method	n_runs	C-index (RSF)
Flchain	CleanSurvival	10	0.89 ± 0.00
Flchain	Optuna	10	0.90 ± 0.00
Flchain	Random	10	0.90 ± 0.01
Rotterdam	CleanSurvival	10	0.91 ± 0.00
Rotterdam	Optuna	10	0.91 ± 0.00
Rotterdam	Random	10	0.91 ± 0.00
GBSG	CleanSurvival	10	0.65 ± 0.00
GBSG	Optuna	10	0.73 ± 0.00
GBSG	Random	10	0.74 ± 0.01

Table 3: Full runtime table for the evaluated settings. Time-to-target is measured as time to 95% of best-observed C-index.

Dataset	Missingness	Mechanism	Method	n_runs	Time to 95%	Final C-index	Pipelines
Flchain	0%	None	CleanSurvival	16	–	0.669	15
Flchain	0%	None	Optuna	50	–	0.656	15
Flchain	0%	None	Random	30	11.70 s (IQR 2.55)	0.672	15
Rotterdam	50%	MAR	CleanSurvival	20	0.16 s (IQR 0.03)	0.836	15
Rotterdam	50%	MAR	Optuna	59	0.15 s (IQR 0.01)	0.829	15
Rotterdam	50%	MAR	Random	20	6.82 s (IQR 1.73)	0.827	15
Rotterdam	50%	MCAR	CleanSurvival	44	2.27 s (IQR 4.68)	0.833	15
Rotterdam	50%	MCAR	Optuna	81	5.19 s (IQR 0.45)	0.829	15
Rotterdam	50%	MCAR	Random	20	5.02 s (IQR 1.55)	0.825	15
Rotterdam	50%	MNAR	CleanSurvival	28	–	0.833	15
Rotterdam	50%	MNAR	Optuna	68	–	0.850	15
Rotterdam	50%	MNAR	Random	30	5.85 s (IQR 2.66)	0.836	15
GBSG	50%	MNAR	CleanSurvival	2	0.10 s (IQR 0.00)	0.727	10
GBSG	50%	MNAR	Optuna	50	–	0.650	15

Table 3: Full runtime table for the evaluated settings. Time-to-target is measured as time to 95% of best-observed C-index.

Dataset	Missingness	Mechanism	Method	n_runs	Time to 95%	Final C-index	Pipelines
GBSG	50%	MNAR	Random	30	3.61 s (IQR 0.00)	0.669	15

2 Convergence Trajectories

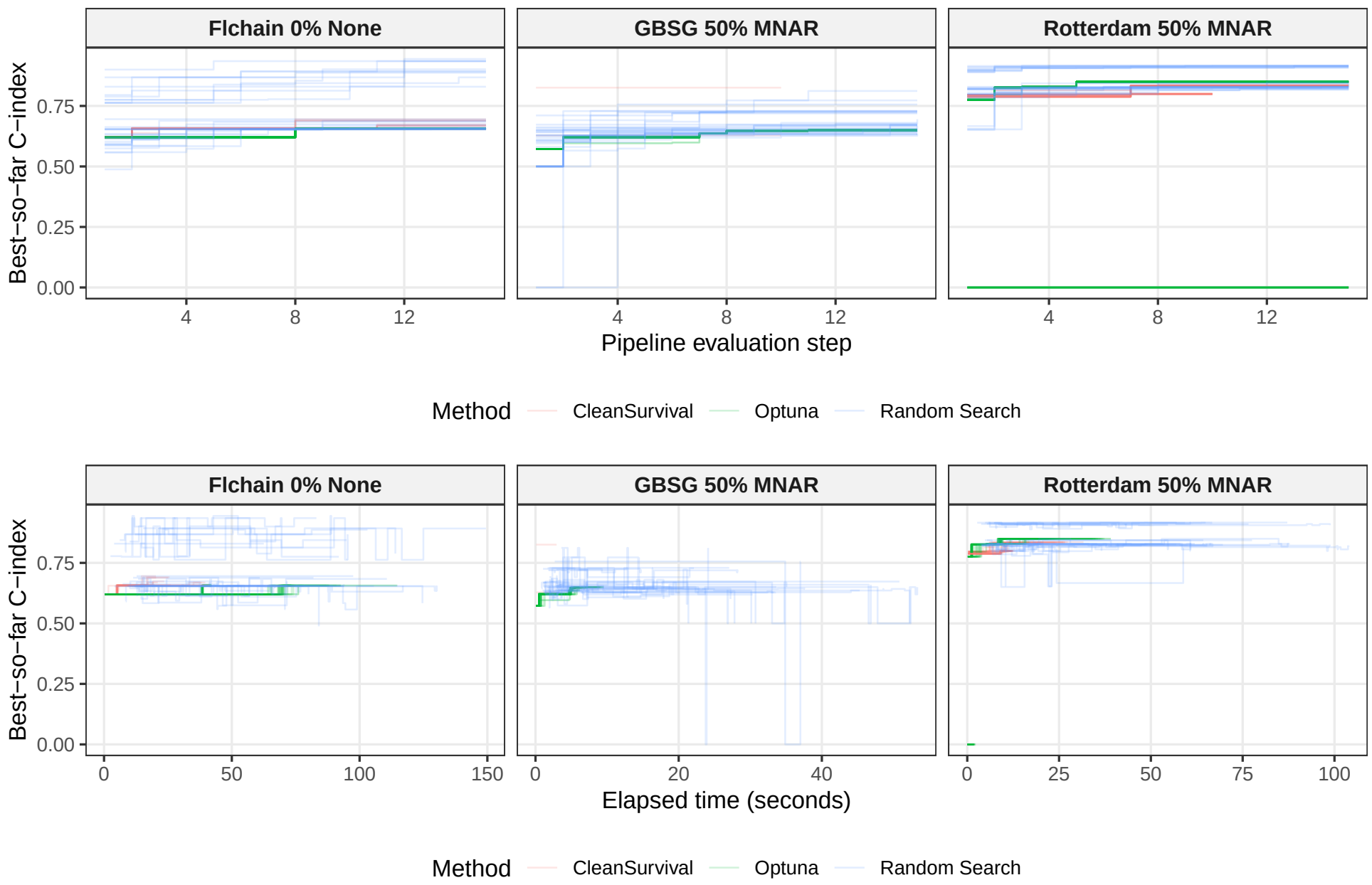

Figure 1: Convergence trajectories across the evaluated benchmark settings. Left panel: best-so-far C-index by pipeline evaluation step. Right panel: best-so-far C-index by elapsed time. Individual runs are shown as semi-transparent traces.

3 Proportional Hazards Diagnostics

Figure 2 shows Schoenfeld residuals for the Rotterdam Cox model after preprocessing with CleanSurvival. Most smoothed residual trends are close to flat around zero, with no clear pattern of severe time dependence. This provides supportive descriptive evidence for the proportional hazards assumption.

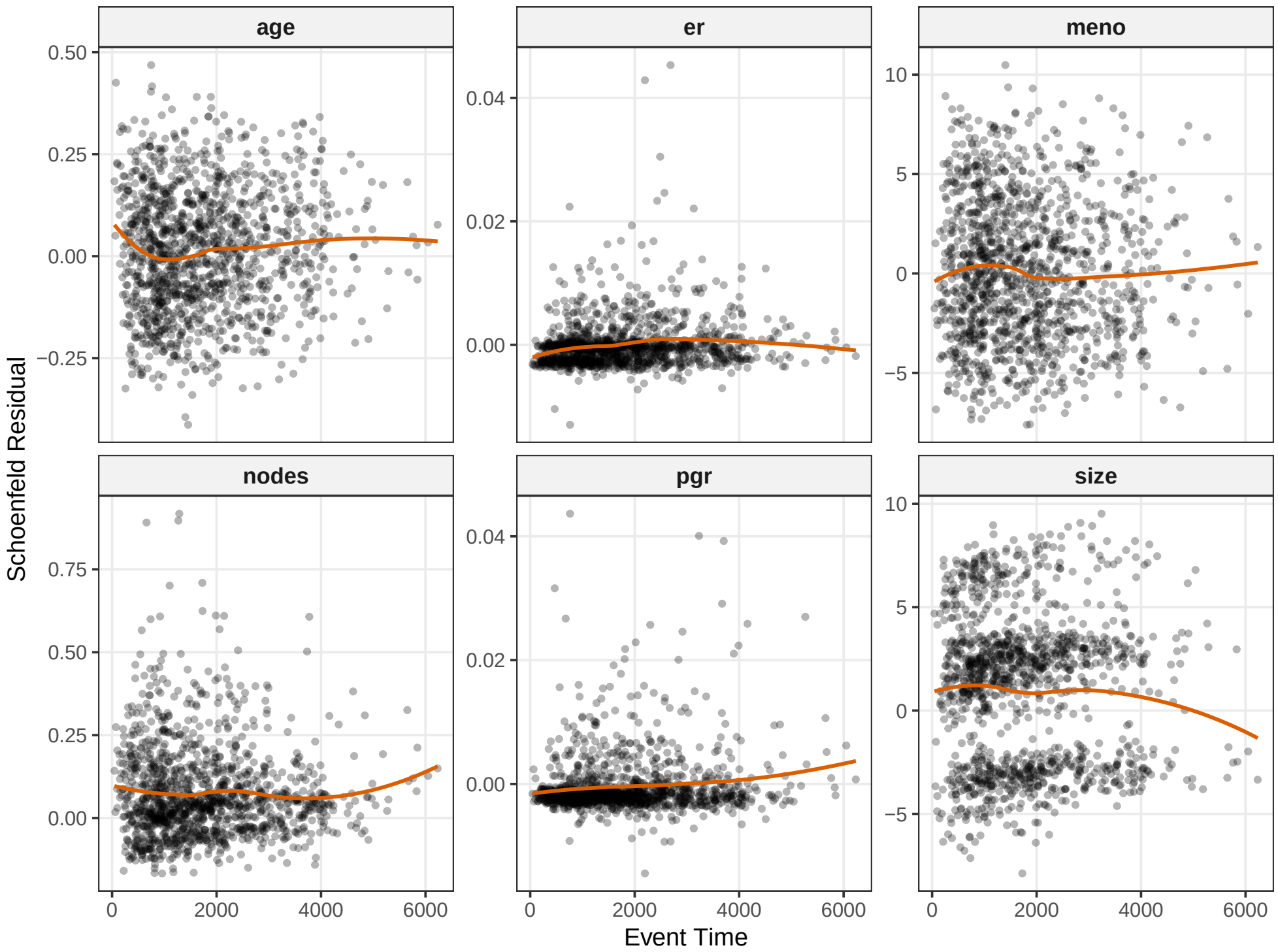

Figure 2: Schoenfeld residuals over time for the optimal Cox pipeline on the Rotterdam dataset. A lack of clear trends indicates general adherence to the proportional hazards assumption.

The smoothing splines in Figure 2 appear relatively flat and near zero for most covariates, suggesting no obvious severe violations of proportional hazards in this diagnostic view. We treat this as descriptive evidence rather than a formal pass/fail criterion.
